# Multiple genome alignment in the telomere-to-telomere assembly era

**DOI:** 10.1186/s13059-022-02735-6

**Published:** 2022-08-29

**Authors:** Bryce Kille, Advait Balaji, Fritz J. Sedlazeck, Michael Nute, Todd J. Treangen

**Affiliations:** 1 Department of Computer Science, Rice University, Houston, TX USA; 2 Human Genome Sequencing Center, Baylor College of Medicine, Houston, TX USA

**Keywords:** Multiple genome alignment, Comparative genomics, Homology, Synteny

## Abstract

With the arrival of telomere-to-telomere (T2T) assemblies of the human genome comes the computational challenge of efficiently and accurately constructing multiple genome alignments at an unprecedented scale. By identifying nucleotides across genomes which share a common ancestor, multiple genome alignments commonly serve as the bedrock for comparative genomics studies. In this review, we provide an overview of the algorithmic template that most multiple genome alignment methods follow. We also discuss prospective areas of improvement of multiple genome alignment for keeping up with continuously arriving high-quality T2T assembled genomes and for unlocking clinically-relevant insights.

**Supplementary Information:**

The online version contains supplementary material available at (10.1186/s13059-022-02735-6).

## Background

The advent of new sequencing technologies has made available hundreds of high resolution vertebrate genome assemblies and along with them the possibility of answering a number of biological questions [1]. As a shining example of the potential of advances in sequencing technologies coupled together with computational methods, in 2022 the complete sequence of a human genome was published [2]. While a single genome alone can yield numerous discoveries, it is the relationships among genomes which hold the key to understanding genomic function and the evolutionary history behind it. These relationships may be small variations, intergenomic distances, ultra-conserved or hyper-variable regions, or structural variation, to name a few [3–7]. Multiple genome alignments are able to accurately represent all of these relationships and more, serving as a rich platform for numerous downstream avenues of comparative genomic analysis.

A useful starting point for discussing multiple genome alignment (MGA) is to first define multiple sequence alignment (MSA). MSA is the method of assigning homology relationships to nucleotides across 3 or more sequences (for 2 sequences, we will use “pairwise” instead of “multiple”), where a set of nucleotides are homologous if they descend from the same common ancestor. These alignments are typically represented by a 2-dimensional array where each row represents an input sequence and each column represents a group of homologous nucleotides. MSA has the strict constraint that the alignment is *colinear*, i.e., each row read from left to right, ignoring the empty columns, must be the original input sequence. As a result, MSA can only capture small indels and point mutations. Finding alignments that maximize homology is a computationally difficult task; maximizing the sum of pairwise matches is NP-Hard in the number of sequences [8–10]. Furthermore, MSA does not model evolutionary events such as inversions, translocations, and the gain or loss of entire genes. In some cases, these sequences are medically relevant and implicated in human disease [11]. Despite the inherent shortcomings, MSA is a critical first step in studying homologous relationships and is a vital precursor to reconstructing a phylogenetic tree accurately [12]; by one estimate it is one of the most studied scientific problems of all time [13].

Following the sequencing of the first full genome in 1995 [14], early methods attempted to perform large-scale MSA on whole genomes. However, the initial release of the human genome in 2000 [15] showed that the same constraints which narrow the solution space of MSA prohibit it from capturing all of the evolutionary events that need to be tracked, which turn out to be common [7, 16–23]. In turn, the task of aligning entire genomes and properly accounting for and coherently reporting these events took on a separate formulation known as MGA. By first finding sets of homologous regions across the input genomes and then aligning them with MSA algorithms, MGA is able to leverage the advances in multiple sequence alignment while still modeling genomic events that MSA does not handle. Indeed, a core problem in MGA is how to correctly find these homologous regions. As MGA consists of finding and aligning homologous regions of the input genomes suitable for MSA, it is no surprise that MGA is inherently at least as computationally challenging as MSA.

Two recent reviews have given a broad background on MGA and described a number methods available for it [24, 25]. Our review will instead focus on MGA algorithms and the implications of the growth in genome data for future development and evaluation of MGA algorithms.

### Evolutionary relationships and their impact on genome alignment

**Fig. 1:**
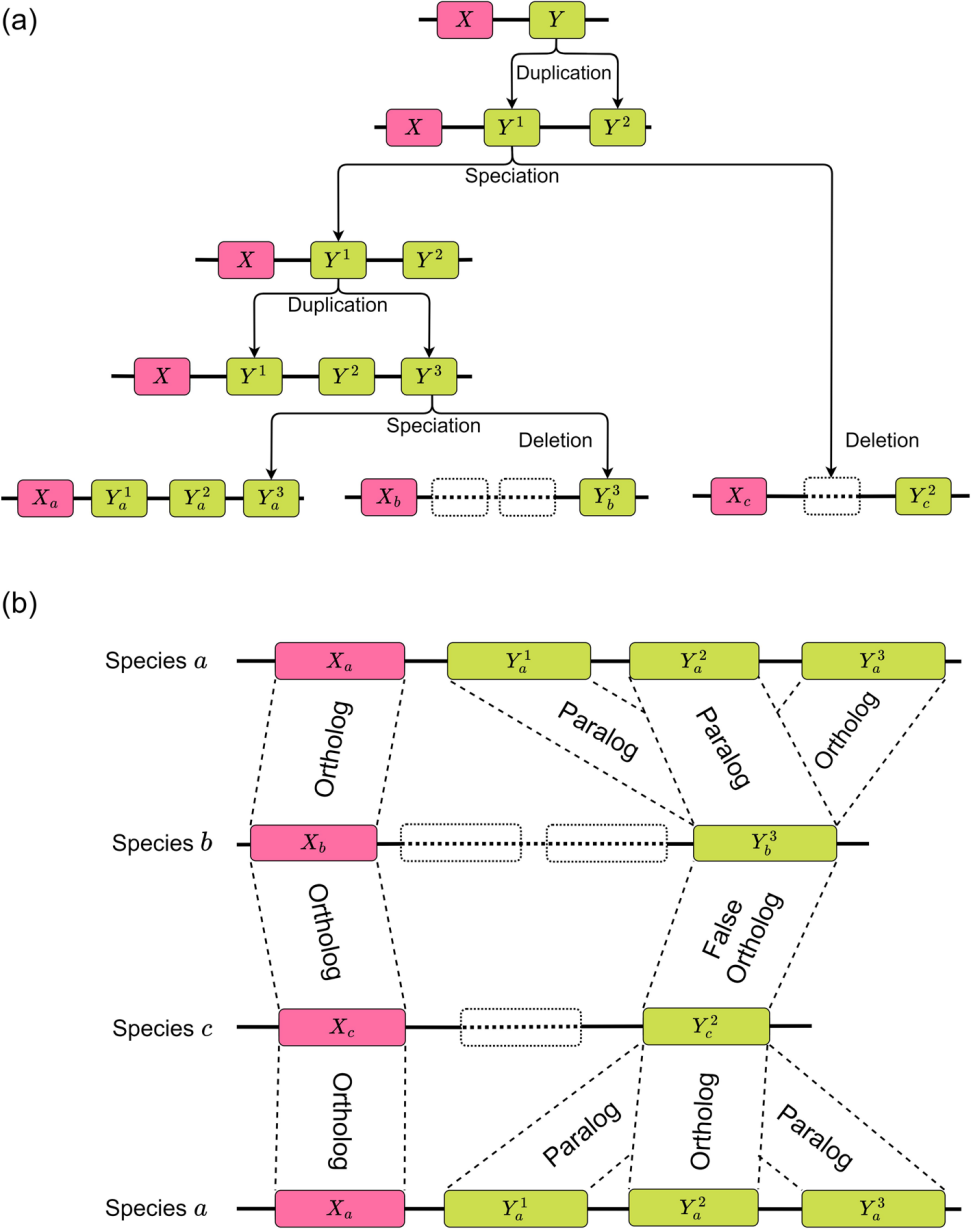
An example of evolution resulting in different classes of homology. In (**a**), deletion and speciation events are depicted, resulting in both orthologs and paralogs. The resulting relationships between all pairs of X segments as well as between all pairs of Y segments are depicted in (**b**). Notably, segments $Y_{b}^{3}$ and $Y_{c}^{2}$ participate in three important homology relationship types. As the most recent common ancestor of $Y_{a}^{3}$ and $Y_{b}^{3}$ is at a speciation event, they are orthologous to each other. Segments $Y_{c}^{2}$ and $Y_{a}^{1}$ on the other hand are paralogs, as their homology is a result of duplication. Finally, $Y_{b}^{3}$ and $Y_{c}^{2}$ are special types of paralogs known as “false orthologs” i.e. paralogous segments all of whose other copies in their respective genomes have since been deleted, resulting in two segments which appear to never have been duplicated. The presence of such false orthologies complicates the problem of core-genome alignment particularly, but also the problem of further categorizing homologies identified through genome alignment. It is worth noting that homology relationships within a single species’ genome do exist, but are not depicted here. For example, $Y_{a}^{1}, Y_{a}^{2}$ and $Y_{a}^{3}$ are all paralogous to each other

After billions of years of evolution, insertions, deletions, substitutions, and rearrangements are ubiquitous to the genomes comprising the Tree of Life [26]. The accurate identification of homologous regions is a cornerstone of comparative genomics [27] and central to enabling the inference of their evolutionary history [28]. Homology identification refers to the identification of regions of common evolutionary history and encapsulates the evolutionary cataloging of each nucleotide within a genome that can span billions of nucleotides. Specifically, homologous regions can be further characterized as orthologs and paralogs [29, 30]. Orthologs are defined as homologous sequences separated by a speciation event, while paralogs are those which are evolutionarily related through a duplication event (Fig. 1). While orthologs are more likely to perform similar functions [31], paralogs are a major force in driving evolution [32]. Inaccurate reconstruction of the underlying gene families can result in erroneous phylogenomic inferences. Further complicating matters, genomes undergo recombination and are subject to multiple evolutionary processes that can radically alter their genomes, including inversions and rearrangements. For a more in-depth discussion on the relationship between genome position and orthology, we refer the reader to [33].

A slight variation of the problem of MGA involves aligning only the “core-genome.” The core-genome of a set of sequences consists of orthologous segments which are shared between all of the sequences [34, 35]. Core-genome aligners like Mauve [36] and Parsnp [37] are often very scalable, as the set of relationships to consider is significantly smaller than in MGA since they do not consider duplicates or orthologies only between a subset of sequences. Core-genome alignment has its own unique set of challenges, though, not least of which is that the more genomes at hand, one finds, the smaller the core-genome gets [38].

Determining whether two homologs are orthologs or paralogs is a challenging problem which remains actively studied [39]. Additionally, the orthology relationship is not an equivalence relationship [30], which complicates the problem of alignment. Further complicating matters, paralogs whose duplicates have been deleted can appear as orthologs due to the absence of duplicates.

### Tracking homologous regions across assembled genomes

As MGA consists of finding and aligning homologous regions of the input genomes; a natural first step is identifying such regions. Pairwise local sequence alignment methods have been the prevailing technique to find and track homologous segments between genomes. First, all pairs of genomes are searched for high scoring local alignments i.e. highly similar segments. These pairwise local alignments are then merged together to identify regions of high similarity across subsets of the input genomes, also known as multiple local alignments.

Pairwise local alignment tools form the foundation for the most widely used global genome alignment methods [40, 41]. However, existing methods that identify and align all homologous nucleotides in two or more genomes have suffered from poor scalability, limited accuracy, or both [42]. While recent progress in the field of alignment has yielded MSA methods that can scale up large datasets while producing accurate alignments [43–45], these methods are “global” and therefore are less applicable when the boundaries of the homologous regions are uncertain.

Handling local sequence similarity within global sequence alignment was previously coined “glocal” alignment (combining global and local alignment) [46], combining local alignments with chaining algorithms as a building block for MGA. Thus, to cope with all of the aforementioned biological and computational hurdles, most state-of-the art MGA methods rely on either progressive alignment [40, 41, 47] built off of “glocal” pairwise sequence alignments and an “aligning alignments” step [48], or divide-and-conquer heuristics [49, 50] to scale with the rapidly increasing set of sequenced and assembled genomes.

Over time, it has become apparent that some local alignment algorithms are better suited for application to homology-finding in MGA than others. While using local pairwise matches between all pairs of genomes to construct alignments that span multiple genomes is very sensitive, the number of pairwise alignments that must be run grows quadratically with the number of genomes, so an efficient local pairwise aligner is paramount. Alternatively, very specific local alignment methods, such as finding sequences shared across all input genomes, are extremely efficient but only reasonable for highly similar sequences. As we will see, the problem of MGA is expanding in all aspects, therefore necessitating the development of new heuristics for large-scale local alignment.

### On the deluge and democratization of high-quality genome assemblies

**Fig. 2:**
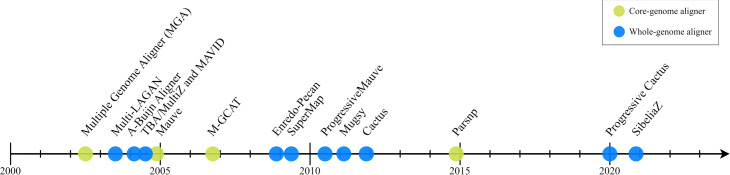
Timeline of MGA tools. The original sequencing of human and mouse genomes spurred the development of a number of multiple genome alignment tools. Following this initial spurt, the next generation of genome aligners (starting with Enredo-Pecan and ending with Cactus) were developed, followed by a 6-year period of silence, with Parsnp being one of the few tools released between 2012 and 2019

Decades of striking technological development have driven huge gains in DNA sequencing throughput with parallel advances in the efficiency of algorithms to assemble the outputs into full-length genomes. That has led to an exponential growth in public genome databases and biologists studying all kingdoms of life are increasingly comparing ever larger numbers of genomes, which means the problem of MGA has changed greatly in character over that time. That being said, the development of algorithms and software for MGA has notably not kept pace (Fig. 2).

**Fig. 3:**
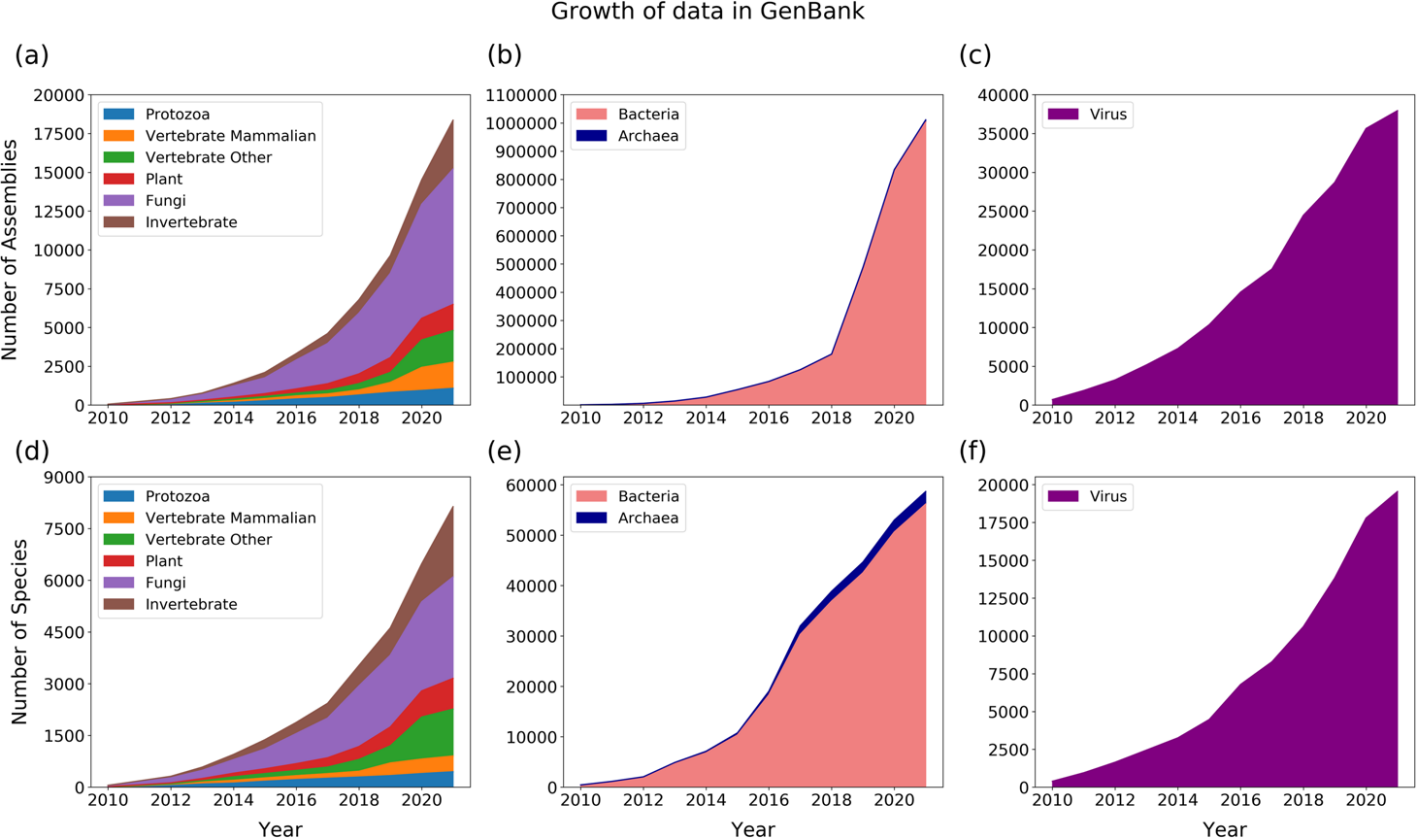
**a**-**c** The number of assemblies available for different groups of eukaryotic species in NCBI. **d**-**f** The number of eukaryotic species with available genome assemblies in NCBI. The emergence of 3rd-generation sequencing technologies and their accompanying assembly algorithms in the early 2010s is largely responsible for the increasing rate of novel eukaryotic genome deposits

The “thousand genome era” is here with respect to vertebrate assemblies, but with nearly 700,000 high quality bacterial genomes available in the European Nucleotide Archive [51] and in excess of 6 million SARS-CoV-2 genomes alone [52], it is clear that the problem of comparing genomes comes in many shapes and sizes, each posing new challenges for MGA (Fig. 3). In 2009, a goal was made to assemble the genomes of 10,000 different species, approximately one from each vertebrate genus [53, 54]. Encompassing the aforementioned goal, the more recent Vertebrate Genome Project [55] seeks to obtain a whole genome sequence of each of the over 66,000 vertebrate species. There are numerous other sequencing projects, some within the Vertebrate Genomes Project such as the Bird 10K project [56] and the sequencing of 44 ruminant genomes [57], and others for non-vertebrate species such as the 10KP plant project [58], the Global Ant Genomics Alliance [59], and the i5k project for arthropods [60]. The Earth BioGenome Project [61] may even subsume them all; a moonshot started in 2018, it aims to sequence all known eukaryotic species over 10 years. All of this is without mentioning human [62], microbial [63–65], fungal [66], and viral [67] sequencing efforts such as the recent deluge of millions of SARS-CoV-2 genomes [52].

### Multiple genome alignment moonshot: the challenge of more genomes

Many of the challenges that hinder MGA approaches are also found in MSA and even local alignment (Table 1) and thus, computationally feasible approaches to MGA often leverage advances from other fields of alignment. One particular lesson from the MSA literature is that the nature of the problem depends on several different properties of the input data, the most obvious of which are sequence length, number of sequences, and overall sequence similarity. For MGA, however, those three properties have particular implications. Sequence length is an obvious difference and many MSA algorithms scale quadratically, or approximately so, in this dimension. Since whole genomes are longer and more structurally diverse than sequences used for MSA, virtually all MGA algorithms use a two-step procedure to break the problem up: first identify highly similar regions across two or more of the sequences known as *anchors* and then use these anchors to identify larger rearrangement-free regions across input genomes known as *locally colinear blocks* (LCBs).

**Table 1:** The many challenges of MGA and potential solutions. As MGA incorporates both local alignment and global MSA, many challenges are shared. While some ideas from MSA have been able to improve the capabilities of MGA, the challenges are by no means solved. Improving runtime, performance across divergent genomes, and accuracy in the presence of repeats still remain important challenges for MGA

Challenge	Solution
Evolutionary distance between genomes increases alignment difficulty [68–70].	Adding closely related species to the input dataset bridges the evolutionary gap between genomes in the input and can increase alignment sizes [71].
Computational costs are currently prohibitive for many large-scale MGAs.	Progressive methods eliminate the need for *O*(*n*^2^) pairwise alignments by only computing a constant number of pairwise alignments at each of the *O*(*n*) nodes.
Low complexity repeats often cause spurious alignments and/or dramatically increase computational cost.	Repeat-masking greatly simplifies the problem of alignment. Indeed, the Cactus GitHub repository states that “genomes that aren’t properly masked can still take tens of times longer to align that those that are masked." [72]
Progressive alignments for whole genomes don’t account for incomplete lineage sorting or horizontal gene transfer, rendering it an incomplete model for both eukaryotes and bacteria.	Divide-and-conquer approaches, similar to those recently used for MSA, could potentially be used to allow sections of genomes to be treated as arising from different phylogenies.
Sequencing error and micro-rearrangements can mask the existence of longer stretches of homologous regions.	Modifications to the genome graphs, similar to those for de-Bruijn graph cleaning, can result in longer, more inclusive LCBs.
The number of pairwise anchors will grow significantly with the number of genomes being compared and the number of anchors present in all genomes will decrease.	Anchoring methods that aim to identify multiple local alignments may bridge the gap between all-pairs anchors and anchors present in all genomes.
While MSA allows for alignment of regions within an LCB, there are limited methods for extending the borders of LCBs.	Multiple alignment extension algorithms, such as the one used in procrastAligner [73], can be employed.

The canonical problem statement of MSA, maximizing the number of pairwise-matching sites, quickly becomes an intractable problem when dealing with more than a few sequences [10] and indeed, pairwise-matching sites are often used as a metric for MGA as well. Two immediate strategies for dealing with this are either to use a set of pairwise alignments for which computation is tractable and resolve the inconsistencies, or to use a heuristic to construct and optimize a local MSA directly. These two strategies underpin the anchor recruitment approaches used in MGA algorithms, as we will see.

One heuristic method often employed is progressive alignment [74], which uses a guide phylogeny to divide the MSA into smaller instances as well as provide an order in which the subset MSAs are merged together. This makes the compute time scale linearly with the number of sequences, but it also means that errors in the process will aggregate as each sequence is added, making these methods respond poorly to more distantly related groups of sequences without regular highly-conserved regions. Progressive alignment has similarly been applied to MGA and as seen in Table 2, nearly half of MGA methods use some sort of progressive approach. Indeed, progressive approaches have even been used to store MGAs [75]. To combat the issues of error propagation, progressive methods in both MSA and MGA have adapted to share information across the subproblems and reduce the introduction of errors [72].

**Table 2:** Multiple genome alignment methods. Tools which only perform part of MGA or are only suited for pairwise genome alignment are excluded. Progressive aligners are italicized. See [24] for a comprehensive list of tools for all subproblems of MGA

Method	Anchor type	Anchor discovery method	Anchor chaining	MSA method
A-Bruijn Aligner [76]	Pairwise alignments	BlastZ [77]	Maximum Subgraph with Large Girth problem [78]	-
Cactus [79]	Pairwise alignments	LastZ [80] and BAR	Cactus alignment filter	Pecan [81]
M-GCAT [82]	MUMs	Compressed suffix graph	Recursive match chaining	MUSCLE [44]
Mauve [36]	MUMs	GRIL [83]	Greedily removes low-scoring LCBs	ClustalW [84]
*MAVID* [85, 86]	*Exon Matches*	*BLAT* [87] *and GENSCAN* [88]	*Smith-Waterman* [89]	*Needleman-Wunsch* [90]
Mugsy [91]	Pairwise alignments	MUMmer [92]	Min-Cut in alignment graph	T-Coffee [93]
*Multi-Lagan* [94]	*Multiple short inexact words*	*CHAOS* [95]	*Longest increasing subsequence*	*Sum-of-pairs/consensus hybrid approach similar to ClustalW*
Multiple Genome Aligner [96]	MEMs	Suffix array	Maximum-weight path in an acyclic graph [97]	ClustalW [84]
Parsnp [37]	MUMs	Compressed suffix graph	Weighted recursive match chaining	libMUSCLE [44]
*Progressive Cactus* [72]	*Pairwise alignments*	*LastZ* [80] *and BAR*	*Cactus alignment filter*	*Pecan* [81]
*progressiveMauve* [98]	*Extending palindromic spaced seeds*	*ProcrastAligner* [73]	*Sum-of-pairs score*	*libMUSCLE* [44]
SibeliaZ [99]	*k*-mers	TwoPaCo [100]	Carrying paths	SIMD partial order alignment [101]
*SuperMap* [102]	*Multiple short inexact words*	*CHAOS* [95]	*Maximum Weight Path Cover*	*Progressive LAGAN* [94]
*TBA/MultiZ* [41]	*Pairwise alignments*	*BlastZ* [77]	*-*	*MultiZ* [41]

For MGA, the problem of scalability in the number and diversity of genomes is exacerbated by the absence of the simplifying assumptions from MSA that restrict the type of mutation events under consideration. It also makes presenting the output more challenging, as a simple row-by-row representation cannot easily display transpositions, inversions or duplications. Methods have emerged for MSA that deal more aptly with large numbers of more diverse sequences, for example by using a divide-and-conquer strategy [103], but most of these have not yet been translated into analogous methods for MGA.

## Breaking down a multiple genome alignment

**Fig. 4:**
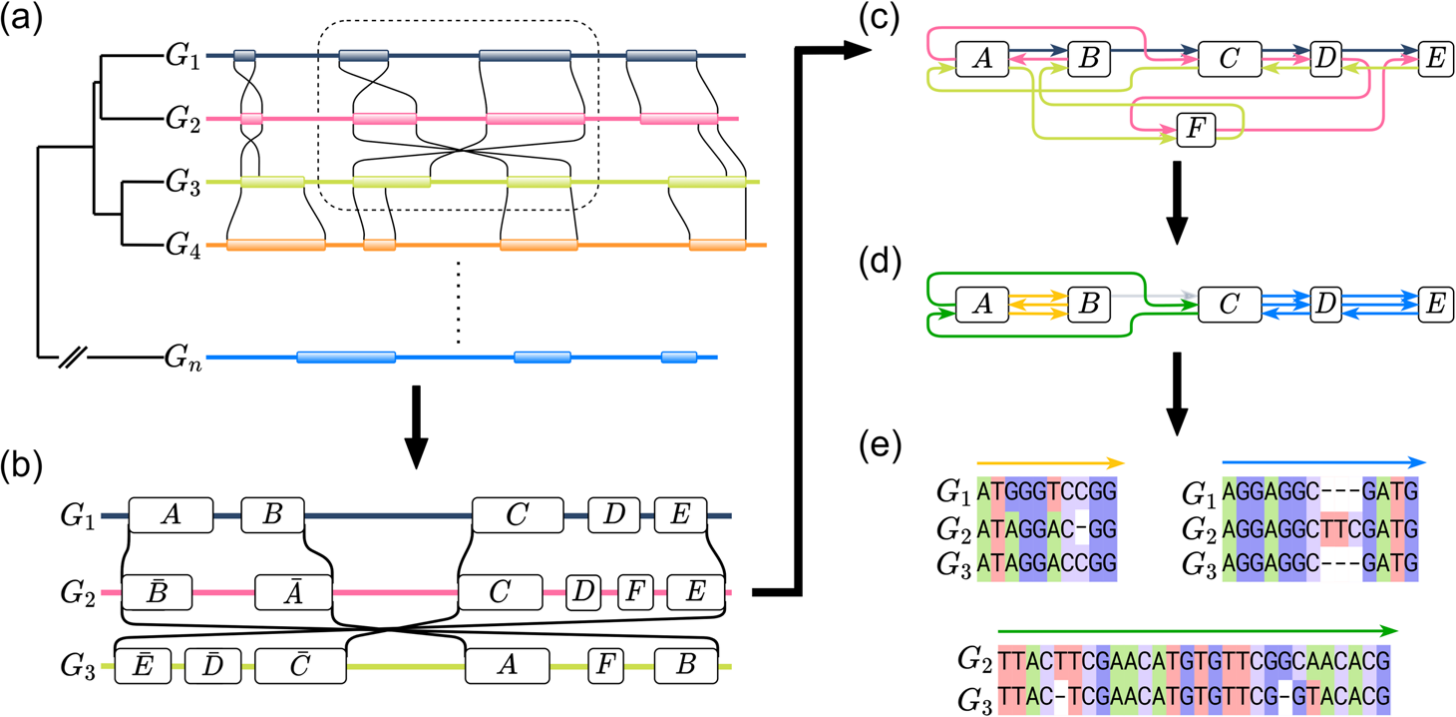
The pipeline of MGA. **a** An example of large-scale genomic rearrangement, insertion, and deletion occurring across a set genomes. The lines denote bounds of homologous segments, and inversions are denoted by crossing lines. **b** Anchors from the section of 3 genomes surrounded by the dotted box in **a**. Once again, the lines between genomes represent homology. The labeled blocks on each genome correspond to anchors, where two blocks with the same label are inferred to be potentially homologous sites. **c** The alignment graph obtained by merging the 3 linear genome graphs in **b**. At this step, the aim of MGA is to find colinear paths in the graph i.e. sequences of anchors which are traversed by a group of genomes in the same order. **d** Often times, the initial set of anchors will be too noisy, containing spurious alignments which prevent the formation of longer, more reliable colinear paths. By removing anchor *F*, the alignment graph becomes much simpler and yields longer colinear paths, where each set of colinear paths is denoted by the color. **e** For each colinear path, MGA tools perform an MSA, yielding a set of sequence alignments which together make up the genome alignment

As noted earlier, the extreme sequence length and presence of genomic rearrangements, gains and losses mean that nearly all MGA programs need to break the problem down first, and can thus be separated into three parts: (1) anchoring, (2) locally colinear block (LCB) identification, and (3) alignment, and in some cases extension, of the LCBs (Fig. 4).

In the anchor recruitment step, the goal is to find well-conserved homologous regions between subsets of the input genomes known as *anchors* (Fig. 4b). These anchors serve as building blocks to identify regions across multiple genomes which share a common ordering of shared anchors are thus colinear and suitable for multiple sequence alignment (Fig. 4c). In order to identify these colinear regions, the blocks are often represented in a graph data structure which algorithms operate on to identify large locally colinear blocks. These blocks are then passed to the final step of MSA and the resulting set of MSAs constitutes the MGA (Fig. 4d). In order to imagine how MGA might begin to cope with more genomes it is helpful to consider the effects of scaling on each of the first two steps separately and in detail.

In the following sections, we will review different approaches to (1) and (2). In many cases, these steps are modular. For example, a recent study took the popular MGA program Mugsy [91] and replaced the MUMmer [92] anchoring step with filtered space word matches [104], yielding a new alignment pipeline which had significantly improved results [105].

### Anchor recruitment

Most anchoring recruitment algorithms can be classified in two distinct ways: pairwise versus multiple anchors, and exact versus approximate. The first classification is selfexplanatory, whether the recruitment identifies anchors that link regions in two genomes at a time or anchors that jointly link at least two of the input genomes directly. The trade-off is that while pairwise anchoring is more sensitive, computing all pairwise alignments scales quadratically with the number of genomes. The second classification is whether exact matching or something more approximate like gapped alignment is used for the anchoring. Again, the trade-off between the two approaches is speed versus sensitivity.

Importantly though, sensitivity is a double-edged sword in MGA. Any anchoring algorithm that emphasizes multiple-genome anchors that span large subsets of the input set of genomes is going to lose sensitivity as the number of genomes and their diversity increases. On the other hand, the increased sensitivity of pairwise alignment is often accompanied by more spurious alignments.

#### Pairwise-Exact: MUMmer

The MUMmer suite is a set of tools for pairwise genome alignment [92, 106–108]. The *mummer* tool from the suite works by first finding all maximal exact matches (MEMs) between a reference genome and a query genome longer than some minimum length *l* by using a suffix array [109]. MEMs which are not unique in either genome can be imprecise for determining homology and also can lead to a quadratic explosion of alignments for high copy number repeats. To accommodate this, MUMmer discards all matches which are not unique in the reference by default.

The MUMmer suite also contains a pairwise aligner, *nucmer*, which uses the intuition that matches which are close together and appear in the same order in both genomes often make up a rearrangement-free homology. First, all matches within distance *g* of each other are clustered together. From each cluster, the maximum length colinear block of matches is extracted. If the total number of nucleotides from all matches within an extracted colinear block does not add up to some parameter *c*, the block is discarded. For each remaining colinear block, the regions between matches as well as the flanking regions of the block are aligned using the Smith-Waterman alignment algorithm [89].

#### Pairwise-Approximate: LastZ

Another widely accepted methodology for computing pairwise genome alignments is the BlastZ method, introduced by PipMaker [110] in 2002 and later improved upon in 2003 [77] and again with its successor LastZ [80]. In order to obtain the anchors, LastZ follows a similar methodology to that laid out by gapped Blast [111], namely by using the X-drop method [112] to compute efficient gapped and gapless extensions from seeds. Given a type of seed, of which LastZ natively supports a few, LastZ finds all pairs of matching seeds across the two genomes. For each pair, a gapped extension is computed, and all extensions scoring below some threshold are discarded, leaving only the high scoring pairs (HSPs). An optional additional step computes a more sensitive seed-and-extend step between colinear anchors.

#### Multiple-Exact: Parsnp

Parsnp discovers anchors for MGA by using a compressed suffix graph (CSG) to obtain maximal unique matches (MUMs) that are present in all input genomes [37]. Parsnp is thus particularly suited for closely related genomes, as the number of MUMs drops off as the sequences diverge. Even one highly divergent sequence is enough to critically reduce the number of MUMs across the entire input and as a result Parsnp provides a first pass filtering step in which any significantly divergent genomes from a user defined reference are discarded.

Following the filtering step, Parsnp constructs a CSG of the reference, a data structure which is able to perform the same MUM finding capabilities of a suffix tree but in a significantly more compressed representation. It should be noted that excluding the filtering step, the selection of the reference will have no effect on the output produced. Each input genome is then streamed through the CSG, resulting in a candidate set of pairwise MUMs. The final set of MUMs used as anchors is the intersection of MUMs obtained from each input genome.

#### Multiple-Approximate: ProcrastAligner

Using the seed-and-extend strategy, ProcrastAligner produces local multiple alignments in a manner which aims to find anchors which span as many of the input genomes as possible. First, ProcrastAligner indexes the location of a set of palindromic spaced seed [113] patterns in order to match both strands simultaneously. ProcrastAligner initiates a priority queue ordered on the multiplicity of each seed. The extension step takes seeds from the queue and computes a gapped extension from each side until a gap of length *w* is incurred. Upon termination of an extension, it is linked to nearby matches. This way, when a less frequent match is popped from the queue, it can avoid computing extensions of linked neighbor regions, as they have already been aligned.

### Graph data structures for alignment

Many alignment algorithms represent the anchors and the sequences between them in graph data structures. Common alignment errors such as short spurious alignments are often signaled by motifs in the graph such as short cycles. By constructing the graph data structure for an alignment, algorithms can identify these motifs and attempt to correct them. Additionally, graph representations provide a straightforward way to both visualize alignments as well as compute them in parallel. In order to describe the locally colinear block construction methods, it is necessary to briefly describe the different graph data-structures that these algorithms operate on. We refer the reader to [114] for a more in-depth discussion of graph data structures for genome alignment.

#### Basic alignment graphs

In the basic alignment graph [10], each node is a genomic segment. For each pair of aligned segments there is an undirected *block-edge* and for each pair of adjacent segments there is a directed *adjacency-edge*. A *block* is a block-edge connected component in the alignment graph. These graphs often serve as the basis for construction of other types of genomic graph data structures.

#### A-Bruijn graphs

In 2004, Pevzner et al. presented the concept of an A-Bruijn graph [78], which merges all nodes in a block-edge connected component in the alignment graph into one single node. While A-Bruijn graphs require additional labeling to recover the original genomes, they have the advantage of representing local multiple alignments with a single node.

#### Enredo graphs

Enredo graphs were introduced as part of the Enredo-Pecan aligner [115] and are similar to A-Bruijn graphs, with the addition that they are able to represent relative orientation of blocks. Let *G*=(*V*,*E*) be an A-Bruijn graph and *G*^′^=(*V*^′^,*E*^′^) be the corresponding Enredo graph. The definition of *V*^′^ is straightforward, we split every vertex in the A-Bruijn graph into a head and tail vertex: *V*^′^={*v*_*h*_,*v*_*t*_|*v*∈*V*}. For each pair of head and tail vertices for a vertex from the A-Bruijn graph, we create a directed block-edge (*v*_*t*_,*v*_*h*_), and the set of adjacency-edges for the Enredo graph is *Ea*′={(*u*_*t*_,*v*_*h*_)|(*u*,*v*)∈*E*}. By adding these head and tail vertices, Enredo graphs are able to provide a sense of orientation to the local alignments from the A-Bruijn graph.

#### Cactus graphs

The Cactus aligner [79] uses a more sophisticated class of graphs, cactus graphs [116], which have the property that every edge is part of at most one simple cycle. We refer the reader to [79, 117] for a more detailed discussion of cactus graphs, but will describe an outline here to provide context for the Cactus LCB construction step. In a cactus graph, the edges are block edges and the nodes in the cactus graph are *nets*, which contain the adjacencies between the connected edges. A fundamental cycle in the cactus graph is therefore a series of consecutive blocks, or local alignments. Additionally, by recursively constructing cactus graphs from the adjacencies within nodes, cactus graphs can easily represent different resolutions of genome alignment.

#### de-Bruijn graphs

For a genome *g* and some integer *k*, we denote de-Bruijn graph [118] as *G*(*g*,*k*). The nodes of *G*(*g*,*k*) are all *k*-mers in *g*, and for every pair of vertices *u* and *v*, there is a directed edge *u*→*v* if the *k*−1 length suffix of *u* is the same as the *k*−1 length prefix of *v*. Therefore, edges in the graph represent (*k*+1)-mers. A de-Bruijn graph of multiple sequences is the union of the de-Bruijn graphs for each individual sequence.

### Locally colinear block construction

Given a set of anchors represented as a graph, the next step is to identify *locally colinear blocks* (LCBs), i.e. regions which share a common ordering of anchors. While the initial set of anchors are sufficient to construct LCBs, they are often noisy and may contain artifacts of micro-rearrangements and assembly error. Consequently, the focus of this step is removing anchors in order to obtain longer, more reliable LCBs. Often times, once the LCBs are obtained methods will repeat the anchoring and LCB construction steps on the regions in-between colinear anchors within an LCB, as these regions are likely to be homologous as well given the high confidence of their flanking regions. In the this section, we provide examples of LCB construction algorithms for different anchor representations.

#### Graph-free LCB construction: ProgressiveMauve

ProgressiveMauve [98] uses MUMs for anchoring, similar to Mauve [36] and Parsnp [37]. However, in ProgressiveMauve the MUMs only need to be present in at least two genomes as opposed to every genome. In order to compute LCBs from this anchoring, ProgressiveMauve works in a pairwise progressive fashion, iteratively working up a guide tree of all genomes. This way, a pairwise alignment only needs to consider MUMs between both genomes. For an iteration of the progressive alignment, ProgressiveMauve processes a node whose two children in the guide tree have been processed. The aim of each iteration is to remove some subset of anchors to maximize a sum-of-pairs score, where the set of pairs is the cartesian product of the descendants from each of the children of the current node. The score for each pair takes into account the number of LCBs shared between the pair as well as the score of the local alignments that make up the LCB. After identification of the LCBs, ProgressiveMauve uses the gapped multiple alignment extension from procrastAligner [73] in order to extend the boundaries of identified LCBs.

#### A-Bruijn graphs: Mugsy

Mugsy [91] represents the initial set of anchors using a basic alignment graph, with the additional feature that it represents alignment orientation in order to capture inversions. Since repeats and duplications can result in multiple segments from anywhere in the same genome being part of the same connected component in an alignment graph, Mugsy uses a greedy depth-first search on each component in order to compute how anchors will be recruited in order to construct the A-Bruijn graph. When the search comes across a duplication or repeat that is not within some predefined distance of its homolog, the copy becomes a new anchor. The resulting set of connected components in the alignment graph is used to construct an “anchor graph,” which shares many of the same features of the A-Bruijn graph. The initial set of LCBs is then calculated by finding simple paths which do not pass through vertices with degree larger than two.

In order to extend the LCBs, Mugsy attempts to remove breakpoints that are artifacts of micro-rearrangements or the initial greedy selection of anchors. To address the former issue, LCBs shorter than some threshold *L* are removed from the graph and any necessary adjacency edges are introduced in their place. Any LCB in the graph containing two anchors *a* and *b* separated by more than *G* from the same genome are disconnected. Such a disconnection is made by using *a* and *b* as source and sink nodes in a min-cut instance [119] on the graph where each edge is weighted by the number of genomes that share that adjacency edge.

#### Enredo graphs: Enredo-Pecan aligner

The Enredo algorithm for creating LCBs in the Enredo graph contains three major steps [115]. In the first, paths in the graph which pass through similar anchor points are merged into single paths which represent the union of the represented alignments. This way, isolated cases of missing or inserted anchors won’t disrupt the ability to construct longer LCBs.

In the second step, Enredo attempts to remove small spurious matches which may result in a scenarios where two potential disjoint LCBs intersect at a spurious anchor. By looking for local multiple alignments whose partitioning into two disjoint alignments results in longer LCBs, the occurrence of such false homologies can be reduced. As a final step, small cycles in the graph, often a result a result of assembly error and duplication, are identified and short anchors from the cycle whose removal results in a larger chain are deleted.

#### Cactus graphs: Cactus aligner

Given a cactus graph, the goal is once again to remove a subset of local alignments in order to improve the obtained LCBs. Cactus provides a mathematical formulation of this problem in terms of the cactus graph. The Maximum weight Cactus subgraph with large chains (MSLC) problem is an optimization problem which seeks to remove as few local alignments as possible while ensuring that all chains in the cactus graph contain at least *α* alignment columns in total [79]. The cactus aligner uses a greedy approach, named the Cactus Alignment Filter, to solve this problem by iteratively removing local alignments from the graph to increase the size of small chains and then adding back in alignments between colinear blocks in a chain. By increasing the cutoff of the minimum chain size at each iteration, the algorithm works up to a graph in which all chains are longer than *α*.

Within a net of the cactus graph, it is likely that there are still rearrangements, albeit smaller, among the adjacencies. In order to resolve these rearrangements, the Cactus aligner seeks to construct another cactus graph from the set of adjacencies within a net. To do this, Cactus uses the Base-level Alignment Refinement algorithm (BAR). BAR uses a modification of the Pecan aligner [115] to align adjacencies within a net that share an endpoint. This set of alignments is likely to contain spurious matches, and therefore the BAR algorithm refines these alignments in order to increase a score determined by a pairwise HMM [120] as well as create consistent alignments. The resulting alignments from the BAR algorithm are then used to construct a cactus graph on the net. This recursion continues and results in a multi-level cactus graph.

#### De-Bruijn graphs: SibeliaZ

SibeliaZ [99] takes a similar approach to its predecessor, Sibelia [121], by first constructing a de-Bruijn graph of the concatenated genomes using TwoPaCo [100], a tool designed specifically for constructing compacted de-Bruijn graphs from many large genomes. With the de-Bruijn graph in hand, SibeliaZ constructs LCBs by iteratively extracting “carrier paths” in the graph. These carrier paths are constructed by starting from a random edge in the graph and iteratively following the heaviest unvisited edge, where the weight of an edge is the number of genomes that it represents. The intuition behind this is that LCBs should share a significant number of *k*-mers, and therefore carrying paths should be able to represent LCBs in the de-Bruijn graph. These walks start at random edges and are terminated when either the score of the path falls below some threshold or there are no valid steps left. The score of a carrying path in SibeliaZ reflects how well the carrying path aligns with the segments it represents, and a carrying path represents a segments if it overlaps with at least *m* nucleotides from that segment.

## Future perspectives

The significant increase in quality and quantity of assemblies has direct consequences for the characteristics of MGA. Long-read sequencing has enabled the closing of countless gaps as well as the correct assembly of repeated regions, which logically leads to more accurate genome alignments. Where short read assembly had over-collapsed and under-collapsed regions, MGA methods would subsequently make erroneous calls of deletions and duplications, respectively. Historically, many methods addressed such errors by identifying and removing small local alignments that correspond to micro-rearrangements. In addition to resolving repeats, complete genomes will also provide adjacency edges along with their sequence labels to alignment graphs which were previously absent or unlabeled. The era of complete genomes also unleashes the possibility of correctly aligning sequences which often elude current methods of analysis. These alignments, both between humans as well as between humans and model-species, can potentially yield new insights into structural variation and human disease [122].

With genomes now being complete and repeat regions resolved correctly, MGA algorithms can focus more on finding accurate local alignments and untangling the resulting net of anchors and less on adjusting for sequencing error. As MGA is a broad field encompassing repeat-masking, local alignment, graph theory, LCB construction, and MSA, it is no surprise that there are a number of avenues for future improvements. In this section, we focus on five main areas that we believe will be key to unlocking future advances in MGA.

### Area 1: Validation and benchmarks

In order to improve the performance of MGA methods, it is critical to develop standardized validation methods which accurately model the problem at hand. The lack of a comprehensive benchmark for MGA methods spurred the Alignathon [123] in 2014. In an effort to evaluate existing methods and provide a usable benchmark, the authors used EVOLVER [124] to simulate two datasets, one representing a closely related set of 4 primates and the other representing a more divergent set of 5 mammals. Methods were evaluated by considering the site level, pairwise precision, and recall of the estimated alignments, but only on a sample of sub-regions of the whole. The Alignathon was a high-quality study and the first of its kind, but the small number of genomes involved has meant diminishing applicability as the availability of data grows. Moreover, an MGA algorithm not only has to be accurate within LCBs at the site level, it must also consider challenges like the extent of the alignment identified and whether it captures the more exotic mutations like translocations and duplications.

Further development of a broader benchmark and a more comprehensive set of evaluation metrics would be useful as new tools are developed for the volume of data now common in MGA. With the new era of T2T genomes comes much higher quality assemblies. Indeed, it may now be worth regenerating the alignathon simulation datasets from the now completed T2T human genome [2, 125], although the most recent whole-genome simulation method, ALF [126], is nearly a decade old. In order to provide a better benchmark for MGA, new genome-wide evolution simulation approaches may also be necessary. Parsnp [37] and SibeliaZ [99] both simulated a bacterial dataset for benchmarking, but there is still no accepted benchmark for aligning thousands of bacterial genomes.

Finally, a direction for validation and benchmarking that may be appropriate is to depart from the paradigm of evaluating by measuring quantitative accuracy with respect to a ground truth. An analogy is the *N*_50_ metric in genome assembly, which is informative about a method’s output but is not properly a higher-is-better measure of accuracy (though it is sometimes liberally applied as such). For MGA, given a biological benchmark of appropriate size such as the over 130 high-quality assemblies already available in the Vertebrate Genomes Project [55], some measures of interest would be running time and fraction of overall genome content aligned in LCBs with at least 90% average nucleotide identity (or a different cutoff if appropriate). These alone would be useful points of comparison for a user approaching an application, and they are likely not the only ones.

### Area 2: Reducing computational requirements

Due to the quadratic complexity of all-pairs anchoring methods, a priority aim of many MGA tools is to reduce the computational cost of anchoring while still maintaining a high level of sensitivity. The utilization of a progressive alignment in Cactus has proved to do just that, enabling an alignment of over 600 amniotes consisting of over 1 trillion base pairs. While the progressive approach of Progressive Cactus lends itself to a linear time complexity with respect to the number of input genomes, it is unclear how much computational resources this alignment required. In fact, the authors of SibeliaZ showed that Progressive Cactus was unable to complete an alignment on 8 mouse genomes from a recent dataset [127] in 1 week on 32 threads [99].

With the advent of extremely powerful GPUs in recent decades has also come GPU adaptations to many alignment algorithms [128–130]. Notably, both MUMmer and LastZ have been fitted for GPU use [131, 132], with the latter improvement yielding a consistent 13x speedup on a vertebrate dataset. While GPUs and large compute clusters, whose use has been made easier by workflow engines such as Toil [133] and Nextflow [134], may provide significant wall-clock time speedups in proportion to the resources available, they are only beneficial to those with the necessary resources. In addition, the speedups may be drowned out by the thousands of genomes to come. It follows that hardware-agnostic algorithmic improvements will be necessary in order to enable accurate alignments of thousands of large genomes.

### Area 3: Improved anchoring

Worthy of its own review, the seed-and-extend approach to local alignment has been shown to be heavily influenced by the type of seed used. LAST [135], a pairwise sequence aligner, showed that seeds which are chosen based on rareness as opposed to length leads to the number of matches increasing linearly with sequence length as opposed to quadratically.

Both MashMap [136, 137] and MiniMap [138, 139] use minimizers [140] in order to select seeds for pairwise genome alignment, but both algorithms are tailored towards mapping long reads to references, as opposed to finding small alignments across divergent genomes which are suitable for anchors. It remains to be seen whether minimizers will break into the space of general MGA. Strobemers [141] and syncmers [142], two recent methods for seeding which aim to be more sensitive than minimizers while still being more sparse than static seeding approaches, are also promising avenues for improving genome anchoring. An additional area of improvement is based on the idea of designing seeds beforehand or allowing seed characteristics to be dynamic, as opposed to selecting an arbitrary set based off a seed rule [135, 143, 144].

As seeding is an approach that is used as a tool for more specific applications, it is necessary to benchmark the seed types for different contexts. For example, Edgar [142] substituted syncmers into both MiniMap2 [139] and Kraken [145] and compared the results. It would be interesting to see a similar but more comprehensive benchmark performed for seeding methods in MGA.

### Area 4: Optimizing LCB construction

It could be argued that the method of representing the graph genome, modifying it, and identifying LCBs in the graph is the core novelty of most modern MGA approaches. Given the prominence of genomic rearrangements and repeats in most genomes, the problem of finding homologous regions boils down to untangling a set of more clear and conserved anchoring regions. There are two likely ways forward in order to increase MGA alignment quality given a set of anchors. The first is to come up with new representations of sequences and their anchors. This has been an approach which has yielded significant advances in MGA, with each of the graph types discussed in this review contributing to major milestones of MGA.

Additionally, LCB construction can be improved by improving algorithms for existing representations of genomes and their anchors. Often, methods for removing anchors to construct better LCBs are posed as mathematical optimization problems. In some cases, such as Mugsy [91], these problems are known to be solvable in polynomial time. For other algorithms, such as Cactus’ MSLC problem [79], the A-Bruijn aligner’s MSLG problem [78], and ProgressiveMauve’s sum-of-pairs optimization [98], there are no known polynomial time optimal solutions and therefore heuristic algorithms are employed. Theoretical guarantees, or at the very least heuristic improvements, are likely to advance the field as well.

### Area 5: Reticulation-aware MGA

Progressive approaches to MSA and MGA have been largely successful in enabling the alignment of larger sets of sequences. That being said, the progressive method it is not without faults of its own. Sophisticated progressive methods which account for global data are still susceptible to propagating early errors up the tree [146]. Additionally, progressive methods require a guide tree which is often calculated with pairwise distances. It has been shown that reordering sequences with very similar distances can result in MSA instability [147]. This instability can be remedied at the cost of runtime by allowing multiple alignments at internal nodes as opposed to strictly pairwise (as in Progressive Cactus). It is also worth noting that most progressive alignment methods assume that all areas of the genome share the same phylogeny and are free of gene flow, both of which is known to be a significant factor in evolution [148–150]. Unfortunately, most studies of the effects of progressive alignment are restricted to MSA. It remains to be studied how these factors impact the performance of progressive methods on MGA.

One intersection between the problem of MGA and MSA is the representation of internal nodes. While using ancestral reconstruction algorithms to replace internal nodes by single sequences is simpler and often more efficient, it has been shown that different methods of MSA can lead to biases in ancestral reconstruction [151]. Another method to solve the subproblems of progressive alignment is by representing internal nodes as alignment profiles. Profile alignment in MSA is an old and well studied problem [152] but is significantly more complex in MGA as the profiles should ideally be able to capture genomic rearrangements and large indels as well. Recent genome alignment methods have shown the promise of representing and aligning genome profiles, and more advances are likely to follow [153, 154].

Future methods for MGA will likely follow the path laid out for them by years of work on progressive and divide-and-conquer approaches to MSA. By horizontally splitting the input genomes up, different regions may be treated with different phylogenies. More sophisticated representations which allow for gene flow may also contribute to correctly identifying and aligning shared regions of distal genomes. Furthermore, a divide and conquer approach similar to SATe [103] may aim to split up the input sequences and iteratively co-estimate and refine the alignments and rearrangements along with the phylogeny, yielding more accurate genome alignments as well as more insightful phylogenies.

## Conclusion

There is a stark contrast between the number of MGA approaches developed in the first decade of the 2000s as opposed to the last 10 years. Indeed, there have only been a few methods published since the Alignathon that comprehensively tackle MGA, notably SibeliaZ and Cactus. It is likely that the increasing arrival rate of high quality assemblies will rejuvenate the effort towards advances in MGA, whether it be improvements to existing methods, new representations of anchors, or novel approaches to the problem as a whole. It is entirely possible the computational methods that are in large part responsible for the deluge of T2T assemblies will help to inspire a new generation of MGA approaches to decipher their evolutionary histories. Regardless, future improvements will be required to help advance MGA and allow it to scale up with the vast number of genomes being ushered in by the T2T era.

## Supplementary Information

**Additional file 1** Review history.

## Data Availability

Data for Fig. 3 was obtain from the taxa-specific assembly_summary.txt files located at https://ftp.ncbi.nlm.nih.gov/genomes/genbank.
