# Supplementary material for: Multiple genome alignment in the telomere-to-telomere assembly era
Source: Genome Biol. 2022 Aug 29;23:182. doi: 10.1186/s13059-022-02735-6 (PMC9421119; doi:10.1186/s13059-022-02735-6)
Supplement: Supplementary file 1 — Additional file 1 Review history. [file 13059_2022_2735_MOESM1_ESM.docx]

**1^st^ round**

**Reviewer 1**

**Is the topic of the article timely and of interest to a wide range of readers?**

Yes

**Is the article well written and presented in a logical way?**

Yes

**Do the authors cover the relevant literature in an accurate and balanced way?**

Yes

**Do the authors provide a useful synthesis of the topic?**

Yes

**Do the authors provide insightful discussion of the future directions for the field?**

Yes: I find the list of future perspectives the strongest part of the manuscript. The open problems referred to as Area 1-5 are relevant and layed out well.

**Do any tables, figures or boxes that are present enhance the manuscript?**

Yes

**Would the manuscript benefit from any additional tables, figures or boxes?**

No: I have no suggestions for additional tables, figures or boxes.

In their manuscript of a review article, Kille et al. describe the steps necessary for computing multiple genome alignments (MGAs) and point out a list of open problems and future perspectives for computing MGAs. The authors identified an area of reasearch that has received less attention over the last decade, possibly due to the abundance of short read sequencing data sets and seemingly more pressing bioinformatics tasks. Nevertheless, multiple whole genome alignment are highly relevant for numerous areas of research, reaching from basic research in biology (phylogenetics) to biomedical applications (conservation scores for interpreting the clinical significance of genetic variation). Their computation remains a challenging task with many open problems.

The strength of the manuscript is in my opinion the part on future perspectives. This part of the manuscript may stimulate future work in this important field of research, which is why I generally encourage the publication of the manuscript. In addition, the manuscript nicely covers a wide range of literature from the field. However, I recommend to improve the 'Background' and 'Breaking down a multiple genome alingment' sections before publication. In its current state the text addresses experts in the field that are already familiar with many concepts, even in sections that define basic concepts. As a review article, it should be intelligible to interested readers who are familiar with related topics only.

Here is a more specific list of points I suggest for improvement:

(When referring to line numbers, I use the numbers printed on the margin, not the actual line numbers.)

1. I find the list of future perspectives the strongest part of the manuscript. The open problems named Area 1-5 are all relevant and layed out well. However, Area 6 does not fit into this list and should be reconsidered. Identification of SVs through MGA is not a future advance of MGA but one of many MGA applications. I could imagine this to go into a section on MGA applications although this is beyond the current scope of the article. To name just one other, already ubiquitous application of MGA, liftover of genomic coordinates between species (e.g. human and mouse) relies on genome alignments, ideally MGAs.

2. I am struggling with the first half of the title "Hello alignment, my old friend". The reason for this is that alignment is a very broad term and exists in various forms, e.g. pairwise vs. multiple, local vs. global, collinear vs. non-collinear, ... Only one such form is Multiple Genome Alignment. Please convince me about the value/necessity of this first half.

3. For a review paper, I am missing some guidance of the reader in the background and 'Breaking down a multiple genome alingment' sections. It remains unclear where the paper is going when reading these parts. Some of it seems like a concatenation of paragraphs written independently of each other without transitions from one to the other, which makes it hard to read.

4. A particular example of the need for reader guidance is the selection of anchor recruitment methods (pages 5-7). The selection seemed arbitrary to me at first since the landscape of pairwise alignment tools is huge. Once I realized that the authors picked one representative per class, the selection made sense. This selection needs to be stated somewhere. In addition, I would prefer a comparative discussion about advantages and disadvantages of these classes (what is computed) instead of or in addtion to a list of algorithmic details (how it is computed) that can be found in original papers.

5. Numerous definitions are somewhat unclear or missing. Here are some examples:

- On page 2, the authors define homology. Next the concepts of in-paralogy, out-paralogy and co-paralogy are mentioned wihtout defining what paralogy and orthology really mean. In my opinion, describing [in|out|co]-paralogy is not very helpful there. For understanding MGAs on a basic level, understanding paralogy and orthology is much more important. For the other concepts, a reference to the paper by Colin Dewey, from where Figure 2 was copied, would be more useful.

- Please explain what 'all-pairwise alignments' are for you on page 3, line 32.

- What does it mean that "genomes are maximally long" on page 4, line 30?

- The definition of anchors on page 5 lines 22-25 is somewhat recursive and needs clarification.

6. Some text seems unnecessarily complicated or implicit. For example:

- The sentence on the first page "We start our review by defining MGA, which necessarily relies on us first defining MSA." This is contradictory, you actually start by defining MSA then.

- On Page 6 in lines 7-9 it remains unclear why you would discard the MEMs which are not unique in the reference genome.

- On page 8 in lines 53-54: What are MUMs that are not present in both genomes in the context of pairwise alignments?

- Please revise on page 14 in lines 26-27 "hard-to-resolve genes are genes with [...] genes". What do the backslashes mean?

7. The authors should proofread the final version of the text. There are both missing and too many occurences of 'of' (e.g. first sentence of main text) and other words (e.g. 'project' on page 4, lines 30/60), inversions of words (e.g. 'and MSA many algorithms' on page 4, line 28) and a number of other mistakes that distract from the actual content of the text (e.g. '"carrier paths" paths' on page 10 or translation -> translocation on page 11).

8. Various smaller points:

- Caption of figure 4: To my knowledge and according to Wikipedia, a variant is defined within one species. In the context of genome alignment (between species), these events are more commonly referred to as rearrangements, or gains and losses of sequence.

- Page 2, line 47: I do not agree that recombination complicates matters for MGA between species. It complicates matters for haplotype phasing and imputation but those are very different tasks.

- I assume you refer to the publication of the human genome in 2001 on page 3, line 43. The technological development of sequencing throughput started already earlier.

- On page 5, line 37: More specific than what?

- On page 5, lines 42-50 where anchor recruitment algorithms are classified as pairwise or multiple and as exact or aligned. A match of two identical subsequences is both an exact match as well as a local alignment of two sequences. Therefore, the second classification should be exact or approximate (match) (instead of aligned). I also recommend rephrasing "something more flexible like an alignment".

- Page 5, line 53: The sentence assumes that multiple-genome anchors always include all sequences, without defining this before. As mentioned later in the manuscript, this assumption is not always true.

- Page 6, line 59/60: What is meant by a constant number of extensions per base pair?

- Page 8, line 48: Parsnp is similar to ProgressiveMauve and not vice versa.

- Page 10, lines 29+34: Do carrier paths start at random nodes or random edges?

- Page 12, line 16: Toil is a workflow engine not a workflow. How can this speedup computation? This citation seems out of context.

- Page 14, line 16: Chromothripsis is where many assumptions of genome aligners, e.g. progressive aligners, break.

- Several references contain '???' and need further attention.

**Reviewer 2**

**Is the topic of the article timely and of interest to a wide range of readers?**

Yes

**Is the article well written and presented in a logical way?**

Yes

**Do the authors cover the relevant literature in an accurate and balanced way?**

Yes

**Do the authors provide a useful synthesis of the topic?**

Yes

**Do the authors provide insightful discussion of the future directions for the field?**

Yes: The manuscript contains 4 pages of Future Directions

**Do any tables, figures or boxes that are present enhance the manuscript?**

Yes

**Would the manuscript benefit from any additional tables, figures or boxes?**

No: The tables and figures are sufficient

Dear Colleagues,

I really enjoyed reading your review. It is very clearly written, comprehensively referenced, and thoughtfully commented.

I really have nothing to add: my only very minor comment is that the terms ortholog and and paralog appear without being defined, which is a pity given you have Figure 3 at hand to illustrate these key concepts.

Excellent work,

Daniel Zerbino

**Authors’ response**

First and foremost, we would like to thank the reviewers for taking the time to read and carefully review our manuscript. We have provided detailed point-by-point responses below.

**Reviewer #1:**

1. I find the list of future perspectives the strongest part of the manuscript. The open problems named Area 1-5 are all relevant and layed out well. However, Area 6 does not fit into this list and should be reconsidered. Identification of SVs through MGA is not a future advance of MGA but one of many MGA applications. I could imagine this to go into a section on MGA applications although this is beyond the current scope of the article. To name just one other, already ubiquitous application of MGA, liftover of genomic coordinates between species (e.g. human and mouse) relies on genome alignments, ideally MGAs.

We thank the reviewer and agree that an applications section is beyond the scope of this review. We have removed Area 6 from the Future perspectives section and added the following sentences to retain the key points.

Background:

Furthermore, MSA is incapable of aligning genomic regions which may be prone to evolutionary events that MSA does not model such as inversions, translocations, and the gain or loss of entire genes. In some cases, these sequences are medically relevant and implicated in human disease.

Future perspectives introduction:

The era of complete genomes also unleashes the possibilities of correctly aligning sequences which often elude current methods of analysis. These alignments, both between humans as well as between humans and model-species, can potentially yield new insights into structural variation and human disease.

2. I am struggling with the first half of the title "Hello alignment, my old friend". The reason for this is that alignment is a very broad term and exists in various forms, e.g. pairwise vs. multiple, local vs. global, collinear vs. non-collinear, ... Only one such form is Multiple Genome Alignment. Please convince me about the value/necessity of this first half.

We agree that the first half of the title is ambiguous. We have removed the quotation and the title is now “

3. For a review paper, I am missing some guidance of the reader in the background and 'Breaking down a multiple genome alingment' sections. It remains unclear where the paper is going when reading these parts. Some of it seems like a concatenation of paragraphs written independently of each other without transitions from one to the other, which makes it hard to read.

We thank the reviewer for pointing out a lack of flow and guidance in the background and “Breaking down a multiple genome alignment” section. We made a number of changes to the background to improve the flow and provide motivation for the problem of MGA as well as the motivation for the in-depth perspective on anchoring and LCB identification.

With respect to the “Breaking down a multiple genome alignment” section, the flow has been improved by addressing comment #4 below.

4. A particular example of the need for reader guidance is the selection of anchor recruitment methods (pages 5-7). The selection seemed arbitrary to me at first since the landscape of pairwise alignment tools is huge. Once I realized that the authors picked one representative per class, the selection made sense. This selection needs to be stated somewhere. In addition, I would prefer a comparative discussion about advantages and disadvantages of these classes (what is computed) instead of or in addtion to a list of algorithmic details (how it is computed) that can be found in original papers.

The reviewer raises a valid point that the landscape of pairwise alignment tools is vast. We feel that the intro paragraph of the “Anchor recruitment” section addresses the rationale behind selecting the methods that we did. In addition, the introduction of the “Anchor recruitment” section briefly discusses the tradeoffs of the different classes.

5. Numerous definitions are somewhat unclear or missing. Here are some examples:

- On page 2, the authors define homology. Next the concepts of in-paralogy, out-paralogy and co-paralogy are mentioned wihtout defining what paralogy and orthology really mean. In my opinion, describing [in|out|co]-paralogy is not very helpful there. For understanding MGAs on a basic level, understanding paralogy and orthology is much more important. For the other concepts, a reference to the paper by Colin Dewey, from where Figure 2 was copied, would be more useful.

We thank the reviewer for pointing out the missing definitions of two critical terms. We have updated the manuscript to include the definitions of orthology and paralogy. In addition, we have removed references to the [in|out|co]-paralogy

Orthologs are defined as homologous sequences separated by a speciation event, while paralogs are those which are evolutionarily related through a duplication event.

- Please explain what 'all-pairwise alignments' are for you on page 3, line 32.

We appreciate the reviewer pointing out a possible point of confusion for readers. We have changed the manuscript to more explicitly describe what “all-pairwise” alignments are.

All pairs of genomes are searched for high scoring local alignments i.e. highly similar short segments. These pairwise local alignments are then merged together

to identify regions of high similarity across subsets of the input genomes, also known as multiple local alignments.

and also later

While using local pairwise matches between all pairs of genomes to construct alignments that span multiple genomes…

- What does it mean that "genomes are maximally long" on page 4, line 30?

The reviewer raises a valid concern here, and we have modified our text

…~~maximally long~~ longer and more structurally diverse than sequences used for MSA…

- The definition of anchors on page 5 lines 22-25 is somewhat recursive and needs clarification.

We thank the reviewer and agree that defining anchors in terms of local multiple alignments is somewhat recursive. We have modified the definition to be in terms of homologous regions across subsets of the input genomes.

In the anchor recruitment step, the goal is to find well-conserved homologous regions between subsets of the input genomes known as anchors (Figure 4b).

6. Some text seems unnecessarily complicated or implicit. For example:

- The sentence on the first page "We start our review by defining MGA, which necessarily relies on us first defining MSA." This is contradictory, you actually start by defining MSA then.

We agree with the reviewer and have replaced the contradiction from the introductory sentence.

A useful starting point for discussing multiple genome alignment (MGA) is to first define multiple sequence alignment (MSA).

- On Page 6 in lines 7-9 it remains unclear why you would discard the MEMs which are not unique in the reference genome.

MEMs which are not unique in the reference genome could result in a quadratic explosion of alignments. If there are *n* matches in the reference and *m* in the query, there are *n*x*m* alignments. We have updated the manuscript to clarify this.

MEMs which are not unique in either genome can be imprecise for determining homology and also can lead to a quadratic explosion of alignments for high copy number repeats. To accommodate this, MUMmer discards all matches which are not unique in the reference by default.

- On page 8 in lines 53-54: What are MUMs that are not present in both genomes in the context of pairwise alignments?

We thank the reviewer for raising a potential point of confusion for the readers. MUMs not present in both genomes won’t be considered in that instance of a pairwise alignment. We have made a minor change to the text that should clarify this point.

This way, a pairwise alignment only needs to consider MUMs ~~that are present in~~ between both genomes.

- Please revise on page 14 in lines 26-27 "hard-to-resolve genes are genes with [...] genes". What do the backslashes mean?

The reviewer raises a valid point that using the backslashes to denote the homologous groups is unclear. We have removed this portion of text due to the first review comment.

7. The authors should proofread the final version of the text. There are both missing and too many occurences of 'of' (e.g. first sentence of main text) and other words (e.g. 'project' on page 4, lines 30/60), inversions of words (e.g. 'and MSA many algorithms' on page 4, line 28) and a number of other mistakes that distract from the actual content of the text (e.g. '"carrier paths" paths' on page 10 or translation -> translocation on page 11).

We appreciate the author for thoroughly reading our manuscript and catching these errors. We have fixed them and scrutinized the revised draft for further mistakes.

8. Various smaller points:

- Caption of figure 4: To my knowledge and according to Wikipedia, a variant is defined within one species. In the context of genome alignment (between species), these events are more commonly referred to as rearrangements, or gains and losses of sequence.

We thank the reviewer for correcting our use of terminology. We have updated the caption of Figure 4 to refer to rearrangements, insertions and deletions as opposed to structural variations.

- Page 2, line 47: I do not agree that recombination complicates matters for MGA between species. It complicates matters for haplotype phasing and imputation but those are very different tasks.

We agree with the reviewer that certain types of recombination don’t necessarily complicate the process of MGA, however others, such as bacterial recombination and nonhomologous recombination, do.

- I assume you refer to the publication of the human genome in 2001 on page 3, line 43. The technological development of sequencing throughput started already earlier.

The reviewer raises a valid point here and we have amended the introduction to fix this mistake.

~~A striking technological development since 2001 has~~ Decades of striking technological development have driven huge gains in DNA sequencing throughput…

- On page 5, line 37: More specific than what?

We appreciate the reviewer’s feedback and have removed the relevant sentence from the manuscript.

- On page 5, lines 42-50 where anchor recruitment algorithms are classified as pairwise or multiple and as exact or aligned. A match of two identical subsequences is both an exact match as well as a local alignment of two sequences. Therefore, the second classification should be exact or approximate (match) (instead of aligned). I also recommend rephrasing "something more flexible like an alignment".

We appreciate the reviewer for catching our mistake. Exact matches and alignments do not represent a dichotomy, as matches are within the space of alignment. We have updated our manuscript to reflect this fact.

Most anchoring recruitment algorithms can be classified in two distinct ways: pairwise versus multiple anchors, and exact versus ~~aligned~~ approximate.

The second ~~dichotomy~~ classification is whether an exact match or something more ~~flexible~~ approximate like a gapped alignment is used for the anchoring. Again, the trade-off between the two approaches is speed versus sensitivity.

- Page 5, line 53: The sentence assumes that multiple-genome anchors always include all sequences, without defining this before. As mentioned later in the manuscript, this assumption is not always true.

We appreciate the reviewer pointing out ambiguity in our definitions. We have added the clarification that multiple genome anchors link more than two of the input genomes directly.

Any anchoring algorithm that emphasizes multiple-genome anchors that span large subsets of the input set of genomes is going to lose sensitivity as the number of genomes and their diversity increases.

- Page 6, line 59/60: What is meant by a constant number of extensions per base pair?

A constant number of extensions means that each base pair will only be explicitly aligned a constant number of times, regardless of the number of sequences or their length. As this is not a crucial point, we have removed it to avoid any potential confusion.

- Page 8, line 48: Parsnp is similar to ProgressiveMauve and not vice versa.

We thank the reviewer for this point. However, we do not claim that ProgressiveMauve and Parsnp are similar as a whole (or vice versa), we only claim that they both use MUMs for anchoring. Furthermore, we do specify that the MUMs in ProgressiveMauve do not need to be present in all sequences as in Parsnp.

- Page 10, lines 29+34: Do carrier paths start at random nodes or random edges?

We thank the reviewer for noticing this mistake and have corrected the manuscript to reflect that the carrier paths start at random edges.

- Page 12, line 16: Toil is a workflow engine not a workflow. How can this speedup computation? This citation seems out of context.

We appreciate the reviewer’s clarification about Toil. It is indeed a workflow engine, similar to SnakeMake or Nextflow, and we have amended the manuscript to reflect that. We believe that the citation fits here, though, as we are discussing improvements to multiple genome alignment methods that hinge on compute hardware, such as GPUs or distributed clusters. We have amended the section to make this point clearer.

While GPUs and large compute clusters, whose use has been made easier by workflow engines such as Toil and Nextflow may provide significant wall-clock time speedups in proportion to the resources available, they are only beneficial to those with the necessary resources.

- Page 14, line 16: Chromothripsis is where many assumptions of genome aligners, e.g. progressive aligners, break.

The reviewer makes a valid point that current MGA techniques, namely progressive ones, will not be able to accurately capture these regions. This section has been removed due to comment #1.

- Several references contain '???' and need further attention.

We thank the reviewer for noticing some issues in the bibliography. It seems that there are some issues with the BMC “@book” and “@incollection” reference types that cause the “???” when a publisher is provided. When using a vanilla latex doc, there are no “???” in the bibliography. In order to amend this, we have had to remove the Publisher field from the “@incollection” class and have converted the “@book” class to “@misc”.

**Reviewer #2:**

Dear Colleagues,

I really enjoyed reading your review. It is very clearly written, comprehensively referenced, and thoughtfully commented.

I really have nothing to add: my only very minor comment is that the terms ortholog and and paralog appear without being defined, which is a pity given you have Figure 3 at hand to illustrate these key concepts.

Excellent work,

Daniel Zerbino

Daniel,

Your feedback is greatly appreciated. We have updated the manuscript to contain these important definitions.

Orthologs are defined as homologous sequences separated by a speciation event, while paralogs are those which are evolutionarily related through a duplication event.
